# Accelerated wound healing induced by spinach extract in experimental model diabetic rats with streptozotocin

**DOI:** 10.1038/s41598-023-42033-0

**Published:** 2023-09-11

**Authors:** Sara Rahati, Mohammad Kamalinezhad, Abdolali Ebrahimi, Mohammadreza Eshraghian, Hamideh Pishva

**Affiliations:** 1 Department of Cellular - Molecular Nutrition, School of Nutrition Sciences and Dietetics, Tehran University of Medical Sciences, Tehran, Iran; 2 Department of Nutrition, School of Medicine, Zahedan University of Medical Sciences, Zahedan, Iran; 3 School of Pharmacy, Shahid Beheshti University of Medical Sciences, Tehran, Iran; 4 Department of Pathology, School of Medicine, Shahid Beheshti University of Medical Sciences, Tehran, Iran; 5 Department of Epidemiology and Biostatistic, School of Public Health, Tehran University of Medical Sciences, Tehran, Iran

**Keywords:** Diseases, Endocrinology, Health care, Medical research

## Abstract

Patients with diabetes often have difficult-to-heal wounds. Spinacia oleracea extract comprises anti-inflammatory and anti-oxidative compounds; this research, therefore, studied the impact of Spinacia oleracea extracts on ulcer regeneration. This study was conducted on 72 adult Wistar rats (200 $\pm$ 20 g). They were randomly divided into six groups of twelve. A: Diabetic group receiving normal saline. B: Non-diabetic group receiving normal saline. C: Diabetic group receiving spinach aqueous extract. D: Diabetic group receiving spinach alcoholic extract. E: preventive group that received aqueous extract for 2 months. F: preventive group that received alcoholic extract for 2 months. Ulcer regeneration, vascular endothelium growth factor, blood sugar, and weight changes were measured on days 3, 7, 14, 21, and 30. Macroscopic investigation of the wounds non-diabetic control group, diabetic group, as well as spinach aqueous and alcoholic extract groups, were compared and there were significant changes (P < 0.05). Pathologic examination in the spinach aqueous and alcoholic extract groups, and nondiabetic group than in the diabetic group revealed significant advances (P <  0.05). On the third and seventh days, Vascular endothelium growth factor detected significant differences between groups (P < 0.05). Results indicate that, in regenerating diabetic ulcers, Spinacia oleracea may be effective. It influences the ulcer structure and speed.

Chronic diabetic ulcer (CDU) is one of the most severe long-term complications of diabetes, which often lasts for months or even years. Several factors contribute to impaired wound healing, including skin damage, diabetic neuropathy, ischemia, infections, inadequate control of diabetes, poor nutritional status, and severe complications^1^. Lower extremity CDU is considered a major health problem and social burden in developed and developing countries^2^ with a global prevalence of 6.3%^3^. About 15% of diabetic patients suffer from lower extremity CDU. These wounds are hard to heal, and they are the major cause of foot or leg amputation among diabetic patients^4^.

There are four stages of wound healing: coagulation, inflammation, proliferation, as well as remodeling^5,6^. The wound gets hypoxic immediately after formation due to blood vessel damage. The inflammatory and cutaneous cells, summoned to the wound, generate an accumulation of growth factors and cytokines^7^. Wound contracture begins when the inflammatory phase of wound healing subsides. Myofibroblasts, fibroblasts, and capillary sprouts rush to the wound, and collagen deposition begins. During the following days, epithelial cells, covering the wound, begin to differentiate and produce keratin^7^. Therefore, the performance of inflammatory and angiogenic factors, along with collagen formation, causes granulation tissue to form. Granulation tissue provides substrates for the formation and growth of epithelium. Therefore, coagulation, inflammatory, and angiogenic factors, along with the formation of collagen, granulation tissue, and epithelium, result in wound healing^7,8^.

Owing to its nutritional value, spinach is one of the mild vegetables compatible with any disposition^9,10^. Spinach is a versatile plant that is consumed in both raw (e.g., salads, smoothies) and cooked forms (e.g., steamed, casseroles, soups). Spinach is primarily composed of water (91.4%), and contains small amounts of protein (2.9%), carbohydrate (3.6%), and fat (0.4%). The lipid fraction is mainly composed of mono- and poly-unsaturated fatty acids (alpha linolenic acid, linoleic acid, oleic acid) and trace levels of saturated fatty acids (e.g., capric acid, myristic acid, stearic acid). Spinach (100-g serving) contains 2.2 g fiber and high levels of magnesium, potassium, and iron that meet 20%, 16%, and 15%, respectively of their RDA. A 100-g serving of spinach contains enough of several vitamins to partially meet or exceed their respective RDA, including vitamin K (604%), β-carotene (a precursor of vitamin A) (188%), folate (49%), and vitamin C (47%), vitamin E (10%). Also, spinach is a good source of antioxidant ingredients including flavonoids (1.5 mg CE/100gr fresh weight), phenolic compounds, lutein, lycopene, and linolenic acid, which have a critical role in scavenging reactive oxygen species and protecting cells from oxidative damage^11–14^.

Spinach contains thylakoid^15^ and various types of amino acids, and studies have shown that its arginine contents in the aqueous and alcoholic extracts are 0.28 and 16.84 mg/100 g, respectively. The glutamine content of the alcoholic extract was 29.13 mg/100 g, but it was not detected in the aqueous extract^16^.

Glutamine has a vital part in inflammation reduction and wound healing. It decreases fat mass related to reduced insulin resistance, tumor necrosis factor α (TNFα), interleukin-6 (IL-6), as well as decreased activity of the c-Jun N-terminal Kinase (JNK) and IκB kinase subunit β (IKKβ); thus, in liver and muscle, glutamine improves insulin signaling^17^. Recently, investigations indicate that glutamine rises collagen synthesis indirectly by increasing the transcription level^10^. Some further investigations reveal that flavonoids and especially kaempferol impose a strong inhibitory impact on glycation of body proteins and collagen, particularly so that, in diabetic patients, they defend the skin^18,19^. Many pieces of research indicate that arginine supplementation speeds up wound healing^19^. Previous studies have shown the anti-inflammatory, anti-proliferative, anti-obesity and antidiabetic characteristics of spinach^14,20–22^.

Considering the above-mentioned impacts and the fact that little research has been carried out on the food effect on wound healing, it was decided to examine "the spinach extract effect on diabetic wound healing in diabetic rats using streptozotocin in an experimental model".

## Material and methods

### Statement

The study was performed in compliance with ARRIVE guidelines.

### Preparation of spinach aqueous and alcoholic extracts

The aqueous and alcoholic extract of spinach was prepared in the department of pharmacology, Shahid Beheshti University of Medical Sciences, Tehran, Iran.

### Preparation of the alcoholic extract

Fresh spinach leaves were put in the open air for 14 days to dry and the dried leaves were ground. The powder (100 g) was poured in a beaker and 96% alcohol (1 L) was added. The top of the beaker was covered with aluminum foil, and it was put in the dark at 25 °C for 48 h. The solution was then filtered using filter paper and concentrated by vacuum evaporation to reach the concentration of interest.

### Preparation of the aqueous extract

The powder (100 g) was poured in boiling water (at 100 °C) and kept in it for 5 min. The solution was then passed through filter paper and put in a water bath to be concentrated.

### Animals

Seventy-two male Sprague–Dawley rats aged 8 weeks (weighing 200 ± 10 g) were obtained from Pasteur Institute (Karaj, Iran) and housed individually in polycarbonate cages in a standard environment (20–22 °C, 50% humidity and 12-h light/dark cycles). The rats were fed standard rat chow and permitted to drink water adlibitum. They were alienated into six groups of twelve rats each randomly.

Group A: diabetic rats received 300 mg/ kg normal saline by gavage for a month.

Group B: non diabetic rats received 300 mg/ kg normal saline via gavage for a month.

Group C: diabetic rats received 300 mg/ kg spinacia oleracea aquatic extract by gavage for a month.

Group D: diabetic rats received 300 mg/kg *Spinacia oleracea* alcoholic extract by gavage for a month.

Preventive group E: consisted of intact rats received 300 mg/kg *Spinacia oleracea* aquatic extract by gavage for a month. Then, they were exposed to diabetes and received 300 mg/ kg *Spinacia oleracea* aquatic extract for a month after ulcer induction.

Preventive group F: consisted of intact rats received 300 mg/ kg *Spinacia oleracea* alcoholic extract by gavage for a month. Then, they were exposed to diabetes and received 300 mg/ kg *Spinacia oleracea* alcoholic extract for a month after ulcer induction.

All experiments were approved by the Animal Care & Ethics Committee of Tehran University of Medical Sciences, Tehran, Iran (Number: 21554). Furthermore, methods were conducted according to the relevant guidelines and regulations.

### Diabetes induction

Diabetes mellitus, after an overnight fast, was induced in groups of rats via a single streptozotocin intraperitoneal (IP) injection (STZ; 60 mg/kg) freshly provided in 0.1 M sodium citrate buffer (pH 4.5, erim gulcon, 2012). However, later it was decided to apply 50 mg/kg for injection owing to mortality with this dose (Streptozotocin (STZ) was bought from Sigma-Aldrich (St. Louis, MO, USA). Rats with fasting blood glucose levels above 250 mg/dl (13.9 mmol/dl) were regarded as diabetic and involved in the experiment. A single dose of IP saline was injected into the non-diabetic rats. On the 3td day after the STZ injection, blood was drawn from the tail vein; a glucometer (Accu check, activate Blood Glucose Meter System, Int. Medical Equipment Diabetes Care, Germany) was applied to measure the fasting blood glucose levels.

### Skin ulcer induction

Employing diabetic rats, a profound skin ulcer model was formed as below. Rats were anesthetized by intraperitoneal injection of xylazine hydrochloride (10 mg/kg) and ketamine hydrochloride (25 mg/kg). Rats back hair was shaved, and by a sterile biopsy punch with a diameter of 6 mm (Biopsy Punch, Kai industries Co., Ltd, Gifu, Japan), a full-thickness wound was generated on the dorsal area that was 1 cm away from the spinal column. The rats were housed alone in separate cages after wound generation. Once ulcer induced in diabetic rats, all groups were exposed to *Spinacia oleracea* aquatic and (or) alcoholic extract, as well as (or) normal saline by gavage for a month.

### Evaluation of healing

The Image J program was used to measure the wounds on days 0, 3, 7, 14, 21, and 30 for macroscopic examination. The healing ratio then was computed by the equation below:

$$\mathrm{healing\, }(\mathrm{\%})=100\times \left(1-\mathrm{wound\, area}/\mathrm{initial \,wound\, area}\right)$$

### Histology

On days 3, 7, 14, 21, and 30 in the Groups, for microscopic assessment, rats were killed randomly, and a tissue wound sample with about 2 mm of the surrounding region was separated for the removal. The samples of tissue were fixed in 10% neutral buffered formalin solution. For histological spots, they were then treated to formulate 5 µm-thick paraffin sections. Via the typical technique of hematoxylin and eosin staining, the formation of granulation tissue, angiogenesis, as well as epithelialization was studied (Supplementary Table A). Trichrome Masson’s staining was used for the collagen strings (which will be stained blue)^23^.

### VEGF evaluation

This was previously described^24^. To assess vascular endothelial growth factor (VEGF), rats from each group were randomly selected and killed on days 3, 7, 14, 21 and 30. Then 10 mL of blood was taken and serum was extracted. VEGF was measured using an ELISA test (CSB- E07363r; Cusabio, Hubei, China).

### Statistical analysis

Normal distribution of data was checked using the Kolmogrov–Smirnov. Data were analyzed employing ANOVA and Tukey Posthoc tests. Besides, data were recorded as the mean ± standard error of the mean. ANOVA test was used to analyze the differences between 6 groups based on quantitative variables, including VEGF and blood sugar. Tukey was applied to make comparisons between a set of means. All statistical analyses were carried out using the Statistical Package for Social Sciences (SPSS) version 18. For statistical significance, all tests were two-tailed with the significance level at P < 0.05.

### Ethics approval and consent to participate

All authors agree to the ethics and consent to participate in this article and declare that this submission follows the policies of *Scientific Reports*. Accordingly, the material is the author’s original work, which has not been previously published elsewhere. The paper is not being considered for publication elsewhere. All authors have been personally and actively involved in substantial work leading to the paper and will take public responsibility for its content.

## Results

### The findings of the granulation tissue index

**Figure 1:**
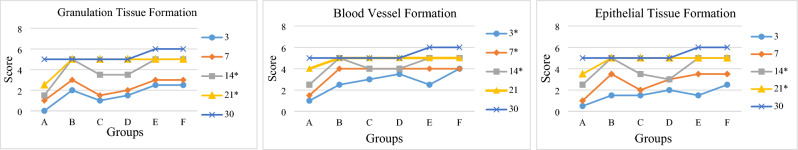
Histological comparison in different groups on different days. (**A**) Diabetic group receiving normal saline (n = 12). (**B**) Non-diabetic group receiving normal saline (n = 12). (**C**) Diabetic group receiving spinach aqueous extract (n = 12). (**D**) Diabetic group receiving spinach alcoholic extract (n = 12). (**E**) Groups of spinach aqueous extract a month before and a month after the induction of diabetes-induced ulcer disease (n = 12). (**F**) Groups of alcoholic extract of spinach a month before and a month after the induction of diabetes-induced ulcer disease (n = 12). Two rats were euthanised at 3, 7, 14, 21 and 30 days from the starting point of wound induction. *P < 0.05.

Figure 1 displays the mean values of granulation tissue on the third, seventh, fourteenth, twenty-first, and thirtieth days in the experimental groups. It should be noted that granulation tissue on different days entailed a normal distribution. Hence, the data were analyzed through a one-way analysis of variance. Using this method, different groups could be compared individually and separately in terms of mean granulation tissue at different days after the intervention. On the fourteenth and twenty-first days, as observed in the table, granulation tissue was significant (p < 0.05). On the thirtieth day, since the wound was completely healed in six groups, all the scores were identical. However, statistical analysis was impossible because there was no statistical difference between the groups. On the third and seventh days, since the mean granulation tissue in the treatment groups was greater than that of group A, the difference was not significant statistically (p > 0.05).

Furthermore, the multiple comparisons of Tukey’s results on the fourteenth day indicated a significant difference statistically between the treatment groups (E and F p < 0.001; C and D p < 0.05) and group A, whereas there was no such significant difference between groups E and F and group B (p > 0.05).

### The findings of blood vessel formation index

As seen, the mean values of new blood vessel formation on the third, seventh, and fourteenth days were statistically significant (p < 0.05). On the twenty-first day, despite the lower mean blood vessel formation in group A compared to the others, this difference was not significant statistically. It is also noteworthy that, according to the results of multiple tests and pairwise comparison of groups, there was a significant difference statistically on the third day between groups F and B (p < 0.05). On the third day, group F showed the maximum blood vessel formation. On the seventh day, there was a significant difference statistically between all groups and group A (p < 0.05). Despite the fact that there was a significant difference statistically on the fourteenth day between all groups and group A, there was a significant difference between groups C and D and groups E, F, and B (p < 0.05) (Fig. 1).

### The findings of the epithelium formation index

**Figure 2:**
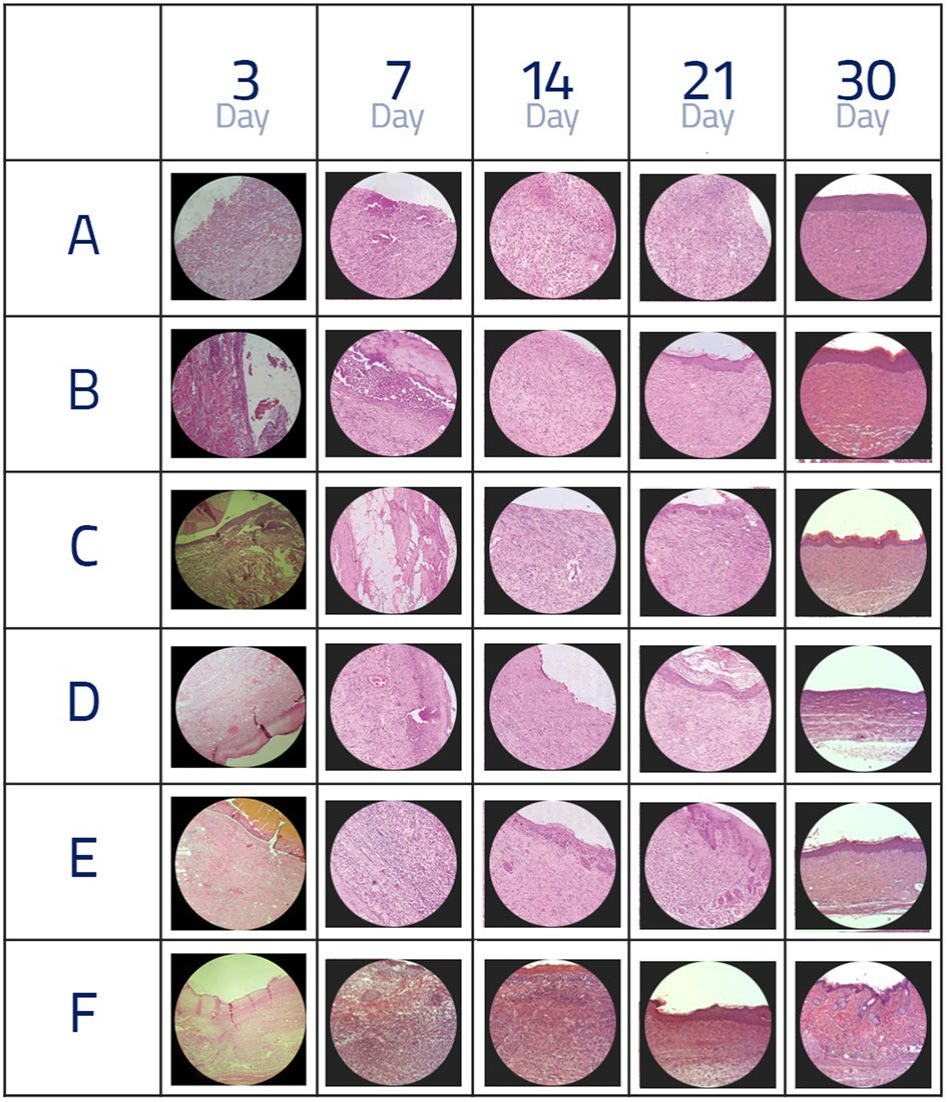
Comparing the formation of granulation tissue, angiogenesis, and epithelium in the study groups on the all days. (**A**) Observation of inflammatory exudate (70%), non-observation of spiky cell migration, and observation of 4–8 vascular sections in the diabetic control group receiving normal saline for 1 month on the third day of the study, (**B**) Observation of more than 40% granulation tissue, 12–14 vascular sections, as well as spiky cell proliferation and highlighting epithelial tissue on the wound edges in the healthy control group receiving normal saline for 1 month on the third day, (**C**) Observation of inflammatory exudate over 60% along with granulation tissue, 15–20 vascular sections, as well as spiky cell proliferation, and highlighting epithelial tissue on the wound edges in the healthy control group receiving aqueous extract of spinach for 1 month on the third day, (**D**) Observation of more than 40% granulation tissue, 15–20 vascular sections, and spiky cell migration on the wound in groups receiving alcoholic extract of spinach for 1 month on the third day, (**E**) Observation of more than 40% granulation tissue, 15–20 vascular sections, and spiky cell migration on the wound in groups receiving spinach extract for 2 months on the third day, (**F**) Observation of extensive granulation tissue, collagen fibers, together with blood vessels perpendicular to collagen fibers, and also more than 20 vascular sections and spiky cell migration on the wound in the group receiving alcoholic extract of spinach for 2 months on the third day.

In Fig. 1, the mean of the epithelium formation on the third and seventh days indicated no statistically significant difference (p > 0.05), while there was a significant difference statistically on the fourteenth and twenty-first days (p < 0.05). On the thirtieth day, the difference was not significant because of complete healing in all the experimental groups. The pairwise comparisons between groups showed that on the third day, only groups F and A had a statistically significant difference (p < 0.05), whereas there was no significant difference between the other groups (p > 0.05). On the seventh day, there was no significant difference between groups F, E, and B and group A (p < 0.05). On the fourteenth day, all groups except D had a statistically significant difference with group A. Moreover, groups D and C showed a statistically significant difference with group B (p < 0.05), while groups F and E had no significant difference with group B (p > 0.05). It is noteworthy that groups E and F and groups C and D showed a statistically significant difference on the fourteenth day (p < 0.05). On the twenty-first day, all the experimental groups showed a significant difference only with group A (p < 0.05) Fig. 2. The formation of granulation tissue, angiogenesis, and epithelium in 6 groups on the all days revealed wound healing.

**Figure 3:**
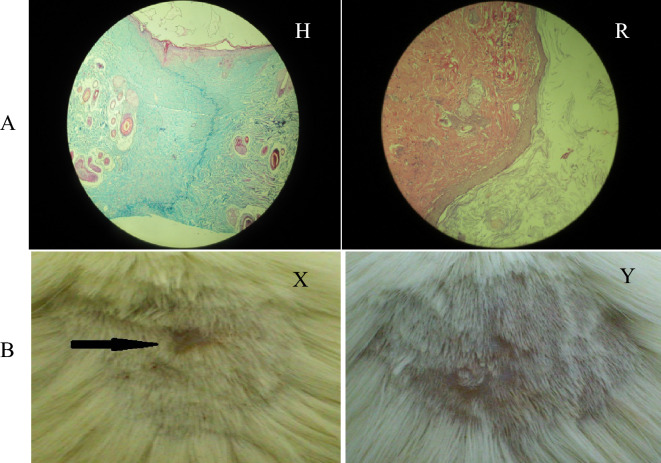
(**A**) Comparing the complete healing of the wound, completely healed and indistinguishable from the surrounding tissue. H: Complete wound healing that is observed as keratin on the wound, extensive collagen accompanied by spindle cells without granulation tissue in the study groups on 30th days (Trichrome Masson’s staining). R: Bridging squamous cells on the wound completely and observing keratin on it, along with absorbing extra and indistinguishable collagen from the surrounding tissue in groups E and F on the 30^th^ day (Hematoxylin & Eosin staining). (**B**) Comparing the percentage of wound healing in the study groups on the 30th day. X: complete wound healing with the scar in study groups on the thirty days. Y: complete wound healing without the scar in E and F groups on the thirty-day study.

Figure 3A, Image H indicated complete wound healing in groups E, F, and B on the 14th day, complete wound healing in groups C and D on the 21st day, and wound healing in group A on the 30th day. Put another way, keratin was fully formed, and granulation tissue was completely fibrosed due to the formation of large amounts of collagen, although the wound was easily distinguished from healthy skin (the surrounding area). However, Image R indicated wound healing in groups E and F on the 30th day when a completely normal piece of skin was observed, and the wound could not be distinguished from the surrounding areas.

### Macroscopic

**Figure 4:**
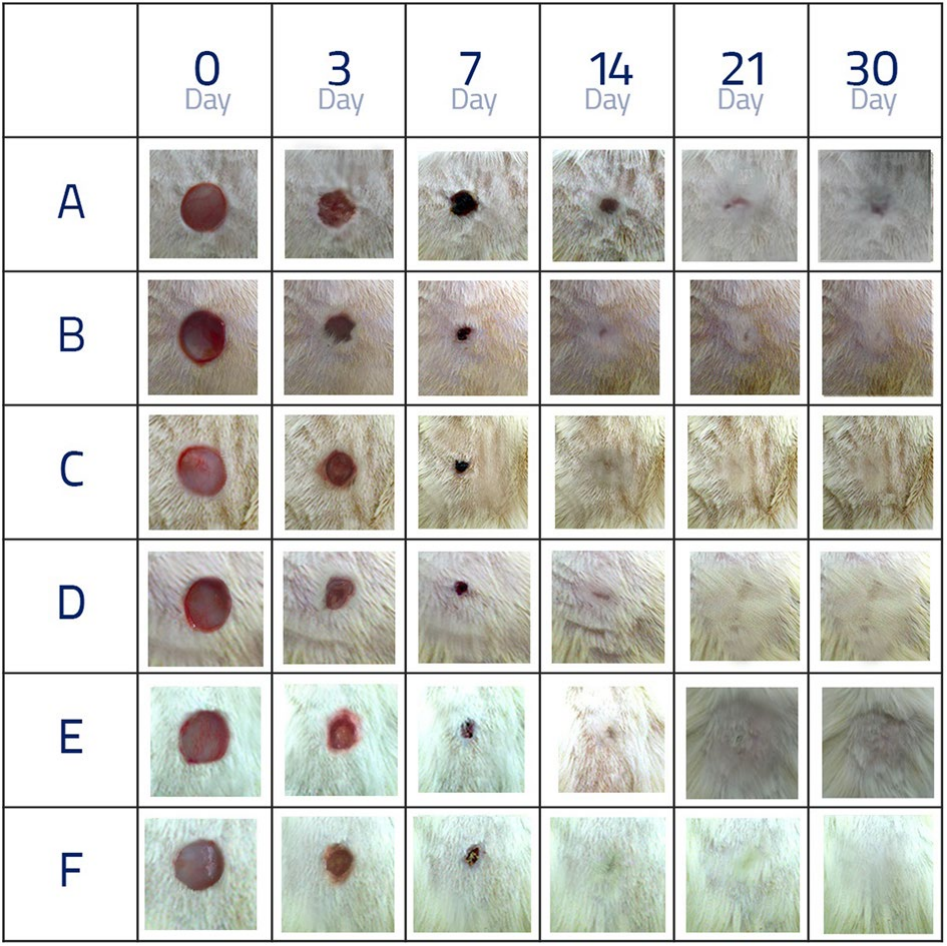
Comparing the percentage of wound healing in the study groups on the all days. (**A**) Diabetic group receiving normal saline (n = 12). (**B**) Non-diabetic group receiving normal saline (n = 12). (**C**) Diabetic group receiving spinach aqueous extract (n = 12). (**D**) Diabetic group receiving spinach alcoholic extract (n = 12). (**E**) Groups of spinach aqueous extract a month before and a month after the induction of diabetes-induced ulcer disease (n = 12). (**F**) Groups of alcoholic extract of spinach a month before and a month after the induction of diabetes-induced ulcer disease (n = 12).

As can be seen, the mean percentage of wound healing on the third (p < 0.05), seventh, fourteenth, and twenty-first (p < 0.001) days indicated a significant difference statistically between the experimental groups. It should be noted that in Table 1, group F had the highest mean percentage of wound healing compared with other groups. Furthermore, groups E and B had a significant difference statistically only with group A (p < 0.05), whereas groups C and D had no statistically significant difference with groups A and B (p > 0.05). On the seventh day (Fig. 4), groups C and D showed a significant difference statistically with group A (p < 0.05), whereas groups F and E had a significant difference with group A (p < 0.05), as well as group B (p < 0.05). There was even a significant difference statistically between group F and groups D and C (p < 0.05). On the fourteenth and twenty-first days, all the treatment groups showed a statistically significant difference with group A (p < 0.05), while there was no significant difference with group B (p < 0.05). On the thirtieth day, owing to complete wound healing in macroscopic terms in all the groups, there was no significant difference statistically between the groups (p > 0.05). The results of Tukey’s test revealed that on the third day, group F had a statistically significant difference with all the groups (p < 0.05) (Table 1).

**Table 1:** Percentage of wound healing (%) comparison in different groups on different days.

Day						
Group (n = 12)	Original wound area (mm^2^)	3	7	14	21	30
A	28.21 ± 2.2	13.0 ± 2.0^b,e,f^	25.0 ± 2.0^b,c,d,e,f^	41.4 ± 5.7^b,c,d,e,f^	80.0 ± 2.9^b,c,d,e,f^	99.0 ± 0.9
B	28.27 ± 2.1	18.5 ± 1.5^a,f^	68.5 ± 1.5^a,e,f^	99.0 ± 1.0^a^	100.0 ± 0.0^a^	100.0 ± 0.0
C	28.23 ± 2.2	15.0 ± 1.0f.	65. 2 ± 2.2^a,f^	93.6 ± 0.33^a^	100.0 ± 0.0^a^	100.0 ± 0.0
D	28.26 ± 2.2	16.5 ± 1.5f.	65.2 ± 2.2^a,f^	93.5 ± 2.5^a^	98.5 ± 0.5^a^	100.0 ± 0.0
E	28.28 ± 2	19.5 ± 1.5^a^	73.5 ± 1.5^a,b^	100.0 ± 0.0^a^	100.0 ± 0.0^a^	100.0 ± 0.0
F	28.25 ± 1.9	24.0 ± 1.0^a,b,c,d^	78.0 ± 2.0^a,b,c,d^	100.0 ± 0.0^a^	100.0 ± 0.0^a^	100.0 ± 0.0
P-value	0.741	**0.017**	**0.0001**	**0.0001**	**0.0001**	0.489

A(a): Diabetic group receiving normal saline. B(b): Non-diabetic group receiving normal saline. C(c): Diabetic group receiving spinach aqueous extract. D(d): Diabetic group receiving spinach alcoholic extract. E(e): Groups of spinach aqueous extract a month before and a month after the induction of diabetes-induced ulcer disease. F(f): Groups of alcoholic extract of spinach a month before and a month after the induction of diabetes-induced ulcer disease. Variables are presented as mean ± SEM. P-value is found by ANOVA test. Two rats were euthanised at 3, 7,14,21 and 30 days from the starting point of wound induction. Significant items with a P value < 0.05 are bolded.

In Fig. 3B, Image X indicated complete wound healing in groups E, F, and B on the 14th day, complete wound healing in groups C and D on the 21st day, and wound healing in group A on the 30th day when the wound was fully recovered, although, the wounded area was totally distinguishable. However, Image Y indicated wound healing in groups E and F on the 30th day. The wounded area was gone and not distinguishable from the adjacent healthy areas. In fact, Fig. 3B confirms Fig. 3A.

### Vascular endothelial growth factor

**Figure 5:**
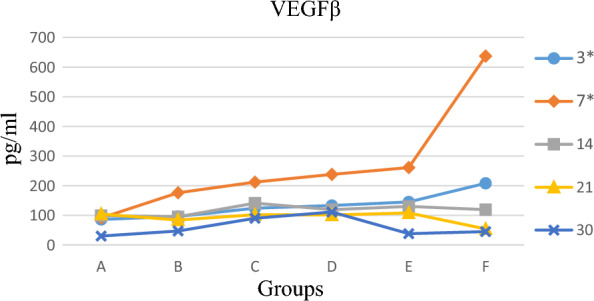
VEGFβ comparison in different groups on different days. (**A**) Diabetic group receiving normal saline (n = 12). (**B**) Non-diabetic group receiving normal saline (n = 12). (**C**) Diabetic group receiving spinach aqueous extract (n = 12). (**D**) Diabetic group receiving spinach alcoholic extract (n = 12). (**E**) Groups of spinach aqueous extract a month before and a month after the induction of diabetes-induced ulcer disease (n = 12). (**F**) Groups of alcoholic extract of spinach a month before and a month after the induction of diabetes-induced ulcer disease (n = 12). Two rats were euthanised at 3, 7, 14, 21 and 30 days from the starting point of wound induction *P < 0.05.

On the third and seventh days, there was a significant difference between the groups, as observed in Fig. 5 (p ˂ 0.001), whereas there was no significant difference statistically between the groups on the fourteenth, twenty-first, and thirtieth days (p > 0.05). Tukey’s multiple tests showed a significant difference on days three and seven between groups C, D, E, and F with group A (p < 0.05). Moreover, on day three, there was a statistically significant difference between the treatment groups (except group C) with group B (p < 0.05). On day seven, however, only group F with group B showed statistically significant differences (p < 0.05). As observed in Fig. 5, the highest mean of VEGF on the third and seventh days was found in group F.

### Blood sugar

Table 2 shows the mean blood glucose; a significant difference is observed on all days (p < 0.01).

**Table 2:** Blood sugar (mg/dl) comparison in different groups on different days.

Group (n = 12)	Day					
	0	3	7	14	21	30
A	528 ± 16.8^b^	512 ± 46.5^b^ ± 512	446 ± 48.0^b^	552 ± 12.5^b,c,d,e,f^	528 ± 7.5^b,c,d,e,f^	572 ± 11.0^b,c,d,e,f^
B	79 ± 5.9^a,c,d,e,f^	72 ± 26.0^a,c,d,e,f^	82 ± 6.5^a,c,d,e,f^	92 ± 16.0^a,c,d,e,f^	86 ± 28.0^a,c,d,e^	88 ± 24.5^a,c,d^
C	508 ± 21.5^b^	513 ± 4.5^b^	442 ± 62.0^b^	337 ± 61.5^a,b^	361 ± 10.5^a,b^	426 ± 18.5^a,b,e,f^
D	487 ± 21.3^b^	501 ± 56.5^b^	478 ± 30.0^b^	409 ± 8.5^a,b^	324 ± 56.5^a,b^	381 ± 32.0^a,b,e,f^
E	517 ± 25.3^b^	504 ± 3.5^b^	539 ± 9.0^b^	395 ± 15.5^a,b^	300 ± 8.5^a,b^	145 ± 14.5^a,c,d^
F	476 ± 21.4^b^	488 ± 20.0^b^	508 ± 71.0^b^	413 ± 70.0^a,b^	244 ± 12.0^a^	115 ± 4.0^a,c,d^
P-value	**0.0001**	**0.0001**	**0.003**	**0.002**	**0.018**	**0.0001**

A(a): Diabetic group receiving normal saline. B(b): Non-diabetic group receiving normal saline. C(c): Diabetic group receiving spinach aqueous extract. D(d): Diabetic group receiving spinach alcoholic extract. E(e): Groups of spinach aqueous extract a month before and a month after the induction of diabetes-induced ulcer disease. F(f): Groups of alcoholic extract of spinach a month before and a month after the induction of diabetes-induced ulcer disease. Variables are presented as mean ± SEM. P-value is found by ANOVA test. Two rats were euthanised at 3, 7,14,21 and 30 days from the starting point of wound induction. Significant items with a P value < 0.05 are bolded.

### Weight

According to Table 3, the mean weight changes during the fourteenth and twenty-first days showed a significant difference statistically (p < 0.05). At the beginning of the work on the third, seventh, and thirtieth days, there was no such difference (p > 0.05). It should be noted that despite the significant increase in the mean weights in groups E and F on the thirtieth day compared to others, the difference was not significant statistically (p > 0.05).

**Table 3:** Weight (gr) comparison in different groups on different days.

Group (n = 12)	Day					
	0	3	7	14	21	30
A	212 ± 3.8	197 ± 10.5	201 ± 3.9	189 ± 3.3^b,e,f^	191 ± 2.5^b,c,e,f^	218 ± 1.5^b^
B	206 ± 3.4	223 ± 14.5	214 ± 5.4	236 ± 10.3^a,c,d,e,f^	250 ± 16.5^a,c,d^	266 ± 35.5^a^
C	210 ± 4.4	207 ± 9.0	200 ± 5.4	208 ± 5.5^b^	223 ± 7.7^a,b,d^	242 ± 5.5
D	210 ± 3.9	183 ± 7.0	202 ± 4.8	201 ± 7.3^b^	196 ± 5.3^b,c,e,f^	215 ± 25.0
E	219 ± 2.8	204 ± 4.0	212 ± 2.8	218 ± 2.6^a,b^	230 ± 6.6^a,d^	256 ± 16.5
F	215 ± 1.5	208 ± 2.0	205 ± 2.2	213 ± 3.2^a,b^	227 ± 4.4^a,d^	248 ± 7.5
P-value	0.109	0.174	0.108	**0.001**	**0.001**	0.141

A(a): Diabetic group receiving normal saline. B(b): Non-diabetic group receiving normal saline. C(c): Diabetic group receiving spinach aqueous extract. D(d): Diabetic group receiving spinach alcoholic extract. E(e): Groups of spinach aqueous extract a month before and a month after the induction of diabetes-induced ulcer disease. F(f): Groups of alcoholic extract of spinach a month before and a month after the induction of diabetes-induced ulcer disease. Variables are presented as mean ± SEM. P-value is found by ANOVA test. Two rats were euthanised at 3, 7, 14, 21 and 30 days from the starting point of wound induction. Significant items with a P value < 0.05 are bolded.

## Discussion

Our results showed that wound healing occurred on the fourteenth day in preventive groups treated with spinach aqueous and alcoholic extract for 2 months, and healthy control group, on the twenty-first day in groups treated with spinach aqueous and alcoholic extract for 1 month, and on the thirtieth day in diabetic control group. Furthermore, the results showed that recovery in groups receiving 2 months of aqueous and alcoholic extract of spinach occurred with greater intensity and speed than other groups, particularly the healthy control group, because the complete wound recovery occurred on this day (the third day), and there was no sign of a wound to differentiate from healthy skin of adjacent areas. Indeed, this reflected the positive effect of receiving spinach extract prior to the induction of diabetic ulcer.

Latef et al. found that wounds in diabetic rats treated with trans-retinoic acid were clotted faster than those treated with the carrier. In histological examination, similarly, there was a denser matrix with spindle cells more abundant in healed wound^25^.

These confirm the results of the present research, owing to, perhaps, the presence of vitamin A in spinach. It has been suggested that vitamin can be useful for the process of healing wounds via stimulating the epithelialization and deposition of collagen by fibroblasts. Vitamin A raises the inflammatory reaction in the wound. It is believed that this increase occurs in inflammatory response due to higher instability of lysosomal membrane, increased input and activation of macrophages, as well as stimulation of collagen synthesis^26^. Also, vitamin C is one of the compounds found in spinach. It raises the content of collagen, fibroblasts, as well as vascular density in the wounds subjected to gamma radiation. Researchers have found that insufficient vitamin C leads to a very low deposition of collagen, slower angiogenesis, together with significant bleeding, resulting in impaired wound healing^27^.

Although the above report shows the positive effect of vitamin C on wound healing and those results are consistent with the ones obtained in the current study, inconsistent results suggest that topical application of ascorbic acid does not affect wound healing of Tympanic membrane, leaving no impact on the histological parameters of wound healing^28^. Vitamin E maintains and stabilizes the membrane mainly by protecting the cell membranes against destruction caused by oxidation^29^. Vitamin K can have a little direct impact on the healing wound for carboxylation of glutamate in blood clotting factors II, VII, IX, and X. However, the lack of vitamin K may lead to the formation of a hematoma in the wound, thus impairing wound healing and making it susceptible to infection. The greatest effect of vitamin K on healing wounds is associated with its hemostatic capacity^29^.

Arachidonic and linoleic acids are polyunsaturated fats essential in the diet of prostaglandin synthesis. Free fatty acids are prostaglandins and phospholipid precursors, deficiencies of which can hinder wound healing in humans and animals, mainly since phospholipids are the crucial components of cell basis membrane, whereas prostaglandins have an important role in cellular metabolism and inflammation^30^.

During the experiments with an animal model, the role of arginine in wound healing become evident. Some mechanisms have been presented to clarify the arginine positive impact on wound healing. The first is that the useful impacts of arginine supplementation on wound healing are alike those associated with growth hormone^19^. The second proposal is that arginine supplementation may heal the wound by stimulating the responses of lymphocytes T (T cell). Obviously, normal wound healing essentially requires T cells^31^. The third proposed mechanism considers arginine as a unique platform for nitric oxide production. Nitric oxide is an extremely reactive radical that may have a key role in wound healing. In fact, Arginine can produce proline essential throughout collagen production, while nitric oxide is effective on angiogenesis, apoptosis, proliferation, and differentiation^32^. Glutamine is one of the main sources of fuel for respiration, supplying nitrogen necessary for the synthesis of amino acids and sugars. Furthermore, it is the precursor of fibroblasts and macrophages in the cell. Also, glutamine is applied as an essential energy source, as well as for lymphocyte proliferation^10^.

Iron is crucial for hydroxylation of proline and lysine, and hence acute shortage of iron may reduce the production of collagen^29^.

Any long-term change in proteins, including collagen and nucleic acids, owing to non-enzymatic glycation (i.e., glucose binding with protein amine groups in an amount proportional to the mean glucose concentration) may also contribute to tissue damage. The initial biochemical changes are reversible through appropriate control over blood sugar. As high levels of blood sugar persist, there are irreversible changes, which can eventually lead to the final products of advanced glycation (AGEs)^33^. Pentosidine is a special marker of glycoxidation. In fact, these end products of advanced glycation alter the physical function of collagen and other proteins. One of the factors delaying wound healing in diabetic patients is the formation of these products and altered functions of proteins, including collagen and their curtailed flexibility. In a research done by Urios in 2007, it was seen that natural flavonoids found in vegetables, such as kaempferol, quercetin, and antioxidants, such as beta-carotene, provide strong inhibitors to prevent the formation of Pentosidine in collagens^34^.

Issazadeh and et al. showed that the alcoholic extract of the Iranian spinach variety had antimicrobial properties due to its high content of phenolic compounds, unsaturated fatty acids, amino acids, terpenes, alkaloids, etc. and hence could be used as an antibiotic^35^. Dehkharghanian also confirmed the presence of polyphenols and flavonoids in aqueous spinach extract^36^.

Moreover, having compared the amount of phytochemicals and bioactive constituents, including phitobatamin, saponins, phenol, tannins, glycosides, flavonoids, steroids, terpenes and cardenolides, in spinach leaf in aqueous and alcoholic extracts of *Spinacia oleracea*, Olagoke et al. observed that the aqueous extract of *Spinacia oleracea* consists of suitable amounts of saponins, flavonoids, terpenes, cardenolides, and phlobatamin, while the alcoholic extract consists of tannins, phenol, glycosides, and steroids^22^ (Supplementary Table B).

Saponin not only enhances re-epithelialization of the wound but also effectively inhibits inflammatory reactions among the early phase, and promotes matrix synthesis throughout the wound healing process^37^. flavonoids worked in all phases of wound healing and activated the intracellular signaling pathways essential for healing to happen. These are important events in the clinical management of diabetic wounds, as they can fight inflammation and stimulate tissue regeneration^38^. Terpenes produce scars with effective tensile strength, function as an adhesive of primary intention, accelerate wound closure, and contribute to collagen deposition^39^. Also, Cardenolides can boost proliferation of fibroblasts result in enhancement in wound healing process^40^.

Tannin showed significant wound healing promotion effect, the mechanism may be due to its function in promotion fibroblast proliferation and migration into wounds of NIH3T3 cell, and also a pretention of antibacterial activities^41^. Studies illustrate that polyphenols from different plant species, take part in different aspects of wound healing, accelerating this process through their antioxidant, anti-inflammatory and antimicrobial properties and their stimulation of angiogenic activities needed for granulation tissue formation and wound re-epithelialization^42–44^. Glycosides have been shown to stimulate fibroblast proliferation and collagen synthesis, increase the production and accumulation of ECM and enhance re-epithelialization by keratinocytes^45^.

According to such mechanisms and the role of various compounds of spinach in wound healing, it can be argued that this combination can provide a synergistic effect because it recovers faster than the control group. Moreover, receiving the extract 1 month before and one month after induction of disease has a stronger effect on wound healing than receiving it for one month, especially in the group receiving an alcoholic extract of spinach a month before and a month after the induction of disease.

One of the basic characteristics of natural wound healing is the formation of granulation tissue. For example, fibrovascular tissue that contains fibroblasts involves collagen and blood vessels, characterizing the recovery reaction. The vascular component depends on angiogenesis, where the vessels appear days after the wound. The capillary growth into the wound provides a conduit for food and other intermediaries of recovery reaction and also metabolite elimination. Inhibition of angiogenesis can impair wound healing^1^. Metabolic disorders can aggravate VEGF in the wound periphery. Ischemia and hypoxia characterize tissue damage, which means that after 5 days, the oxygen pressure in the wounds is 6–7 mm Hg. These values range from 45 to 50 mm Hg in normal tissues. Angiogenic can restore tissue perfusion, reestablish microcirculation, and raise oxygen pressure to 30–40 mm Hg^46^. Therefore, hypoxia increases the expression of VEGF in monocytes, besides other cell types, such as fibroblasts, keratinocytes, myocytes, and endothelial cells^47^. Hypoxia may enhance tissue expression of VEGF and its receptors that, in turn, helps angiogenic reaction. Hypoxia provides VEGF expression through factor-1 alpha, which is induced by hypoxia^48^. The VEGF expression curve is parallel with the hypoxia curve, followed by endothelial cells migrating toward areas with the highest oxygen deficiency. The macrophage can maintain the curve since they survive in areas with low oxygen pressure. Indeed, the hypoxic tissue curve is essential for angiogenesis in wound healing, and the elimination of this curve can inhibit capillary growth^46^.

Our results showed that all study groups except the control had a significant difference statistically with the diabetic group on the third and seventh days. According to the mechanism mentioned above in which VEGF plays a role in wound healing, it can be stated that maximum hypoxia in the affected area in the treatment groups and control were found on the third and seventh days. This is because the VEGF concentration later began to decline, representing a decrease of hypoxia in the affected area through the role of VEGF in stimulating angiogenesis and then compensating the lack of oxygen. As stated earlier, the hypoxic curve is parallel to VEGF one, and when the hypoxia curve declines, its concentration curtails. On the other hand, since the increase in VEGF and angiogenesis occur during the proliferative phase, the results indicates that wound healing in these groups go through the inflammatory phase after three days and step into the next phase of proliferation similar to wound healing in the control group, while a major cause of delay in wound healing in diabetic patients is the prolonged inflammatory phase. This is confirmed by the results of the diabetic group, where the highest level of VEGF is found on the twenty-first day. Another interesting point is the data on group F. As shown in the tables, group F achieves the highest mean on the third and the seventh days, and it manages to make a stark distinction from other groups and even group E on the seventh day. These results indicate that receiving alcoholic extracts of spinach one month before and then one month after the induction of disease and wound leads to maximum effectiveness in increasing VEGF.

According to the results, on the fourteenth, twenty-first and thirtieth days, all the treatment groups showed a significant difference with the diabetic control one. However, the noteworthy point was that there was not a statistically significant difference between preventive groups and healthy control group. The mean blood glucose on the thirtieth day in preventive groups dropped from 250 mg/dl as a marker of diabetes in rats to 145 and 115 mg/dl, falling within the non-diabetic range. This proved the hypothesis concerning the effectiveness of aqueous and alcoholic extracts on blood glucose in diabetic rats on the fourteenth day. Two previous studies focused on the impact of aqueous and alcoholic extracts of spinach on blood glucose in diabetic rats. In a research performed by Kumar et al. in 2010, the anti-diabetic effects of alcoholic extract of spinach were revealed when used at 100 mg/kg for fifteen days^49^. Nevertheless, Gomathi observed the effects of spinach in lowering blood glucose at a dose of 200 mg/kg and 400 mg/kg of aqueous and alcoholic extracts on the twelfth day; this was confirmed by the results of the present study^50^. Moreover, Gomathi found some improvement in the beta cells of the pancreas in the spinach-receiving groups. Spinach decreases serum glucose through inhibiting intestinal α-glucosidase activity, which reduces the digestion and absorption of disaccharides. On the other hand, differentiation of 3T3-L1 pre adipocytes which is a useful model for studying insulin action was increased in the presence of spinach extract, showing its insulin-like and insulin-sensitizing actions^51^. Also, spinach thylakoids can increase the secretion of GLP-1, which is an incretin hormone and induces insulin secretion^20,52^. The effects of spinach and thylakoids on insulin have been very inconsistent in previous studies^53–56^.

The decisive role of insulin lies in inhibiting the breakdown of adipose tissue reserves of triglycerides in NEFA. Fatty acids are the main substrates of ketosis in the liver. Hence, insulin functions as a major ketosis regulator by controlling blood levels of fatty acids and other effects. Insulin deficiency or resistance can leave extensive metabolic impacts. As carbohydrates are not applied and may not be deposited in the liver, there are excess blood glucose levels. Meanwhile, the cells are not able to consume glucose, where insulin deficiency transforms glycogen, fat, and protein into glucose. In the absence of glucose, however, insulin is not consumed, thus aggravating the blood sugar levels. Protein synthesis curtails muscle protein transformation into glucose, which leads to muscle weakness and muscle tissue degradation as the breakdown of fat causes weight loss^57^.

In an investigation to assess the anti-diabetic effect of spinach on alloxan diabetic rats, spinach extract at a dose of 400 mg/kg managed to leave a beneficial effect on weight gain by the twelfth day^50^. Similarly in the current study, it was expected that receiving spinach extract could, on the fourteenth day, improve weight gain in diabetic rats under treatment; however, the results showed that, on this day only, the preventive groups had a significant difference with the diabetic control group, while it was not the case in one-month groups. On the twenty-first day preventive groups showed a statistically significant difference with the diabetic control group, while there was no such statistically significant difference concerning the healthy control group. Conversely, groups C and D showed a significant difference with the healthy control group. Similarly, group C indicated a statistically significant difference with diabetic control one. The results on the thirtieth day, however, contrasted those above since there was no difference between the treatment groups and those of the diabetic and healthy control. This might have occurred due to the adaptability of the diabetic rats in the control group with the disease rather than the hypothesis of ineffective extract in the treatment groups. As the results for blood sugar suggest, the lowered blood sugar began on the fourteenth day, indicating effective compounds found in spinach, such as vitamins, antioxidants, and flavonoids. Enhanced insulin secretion facilitated the entry of glucose into the cells. This, in turn, prevented damage breakdown of structural proteins to a certain extent and improved weight gain. It was, unlike Gomati’s study in which the effectiveness of spinach on weight gain was observed on the fourteenth day, therefore, revealed that day twenty-one achieved the effect on weight because it was the single day on which groups E, F, and C showed a significant difference with the diabetic control group. The seven-day delay in observing the effect of spinach on weight between the current study and Gumati could be associated with the administered dose of extract, which was 300 mg/kg in the current study and 400 mg/kg in the Gumati study, respectively.

## Conclusion

According to the results obtained in this study, the aqueous and alcoholic extracts of spinach can improve diabetic wounds, VEGF, blood glucose, as well as partly weight loss in diabetic rats, and shorten the recovery time. This effect can be far more extensive when the extract is administered one month before the incidence of disease and one month after the induction as compared to one-month administration of the extract. This represents the preventive role of spinach extract in wound healing before the onset of the disease. Moreover, it can function more intensely in the group receiving two months of alcoholic extract as compared to the group receiving two months of aqueous extract. This may be due to the higher antioxidant capability of alcoholic extract as compared to the aqueous one. Indeed, the number of phenolic compounds and glutamine in alcoholic extracts tend to be higher than those in aqueous ones. Therefore, the administration of spinach alcoholic extract one month before the incidence of diabetes and then one month after the incidence could improve wounds in diabetic rats. Therefore, Spinacia oleracea may be efficient in regenerating diabetic ulcers. It influences the ulcer structure and speed and shortens the healing time.

## Limitation

To our knowledge, this is the first study to show the effect of spinach extract on diabetic wound healing enhancement which has provided novel data. However, there are some limitations on how the results are interpreted. Lack of sufficient facilities and budget constraints in order to analyze aqueous and alcoholic extracts of spinach were the major limitations of this study. In addition, the small sample size and lack of access to a tensiometre to measure wound tensile strength were other limitations of this study.

### Supplementary Information

Supplementary Tables.

## Data Availability

The datasets used and/or analyzed during the current study are available from the corresponding author on reasonable request.

## Abbreviations

AGEs: Advanced glycation end products
ANOVA: Analysis of variance
CDU: Chronic diabetic ulcer
IKKβ: IκB kinase subunit β
IL-6: Inter leukin-6
JNK: C-Jun N-terminal kinase
NFκB: Nuclear factor κB
SEM: Standard error of the mean
STZ: Streptozotocin
TNFα: Tumor necrosis factor α
VEGFβ: Vascular endothelium growth factor
