# Supplementary material for: Accelerated wound healing induced by spinach extract in experimental model diabetic rats with streptozotocin
Source: Sci Rep. 2023 Sep 11;13:14933. doi: 10.1038/s41598-023-42033-0 (PMC10495437; doi:10.1038/s41598-023-42033-0)
Supplement: Supplementary file 1 — Supplementary Tables. [file 41598_2023_42033_MOESM1_ESM.pdf]

Accelerated wound healing induced by spinach extract in experimental model diabetic rats with streptozotocin

Sara Rahati[1,2], Mohammad Kamalinezhad[3], Abdolali Ebrahimi[4], Mohammadreza Eshraghian[5], Hamideh Pishva[1]

[1] Department of cellular - Molecular Nutrition, School of Nutrition Sciences and Dietetics, Tehran University of Medical Sciences, Tehran, Iran.

[2] Department of Nutrition, School of Medicine, Zahedan University of Medical Sciences, Zahedan, Iran.

[3] School of Pharmacy, Shahid Beheshti University of Medical Sciences, Tehran, Iran.

[4] Department of Pathology, School of Medicine, Shahid Beheshti University of Medical Sciences, Tehran, Iran

[5] Department of Epidemiology and Biostatistic, School of Public Health, Tehran University of Medical Sciences, Tehran, Iran.

Correspondence to

Hamideh Pishva, Associate Professor, Department of cellular- Molecular Nutrition, School of Nutrition Sciences and Dietetics, Tehran University of Medical Sciences, Tehran, Iran.

Supplementary table A: Microscopic evaluation and scoring of tissue factors

Index / Score	0	1	2	3	4
Formation of new epithelium	No thickening of the edges or proliferation of prickle cells	Proliferation of prickle cells and epithelial tissue prominence on wound edges	Migration of the prickle cells to wound surface	Formation of a complete bridge over the wound by the prickle cells	Keratin on wound surface.
Angiogenesis	No blood vessel	4-8 blood vessel cross-sections	12-15 blood vessel cross-sections	15-20 blood vessel cross-sections	More than 20 blood vessel cross-sections
Formation of granulation tissue	More than 70% inflammatory exudates	More than 60% inflammatory exudates	More than 40% granulation tissue	Extensive granulation tissue together with collagen strings and blood vessels perpendicular to collagen strings	More than 80% complete tissue

At suggested by the pathologist, we gave the score of 5 for complete wound healing and 6 for complete wound reconstruction so that the wound site could not be differentiated from its surrounding areas.

Supplementary table B: Phytochemical Screening of Spinach Leaf [1].

Parameter	Water	Ethanol
Saponins	++	--
tannins	--	++
Phenol	--	+
Flavonoids	+++	--
Glycosides	--	++
Steroids	--	+
Terpenes	+++	--
Cardenolides	+	--
Phlobatamin	+	--

Legend: +++ (Much abundant); ++ (Less abundant); + (Minute); - (Absent)

1. Olagoke O. Phytochemical analysis and antibacterial activities of spinach leaf. Am J Phytomed Clin Ther Vol. 2018;6(2):8.
